# TECHNOLOGY ATTITUDES, PROFICIENCY, AND USAGE PATTERNS AMONG OLDER ADULTS WITH A COGNITIVE IMPAIRMENT

**DOI:** 10.1093/geroni/igae098.2906

**Published:** 2024-12-31

**Authors:** Xin Yao Lin, Walter Boot, Wendy Rogers, Neil Charness, Sara Czaja

**Affiliations:** Weill Cornell Medicine, New York, New York, United States; Weill Cornell Medicine, New York, New York, United States; University of Illinois Urbana-Champaign, Champaign, Illinois, United States; Florida State University, Tallahassee, Florida, United States; Weill Cornell Medicine/Center on Aging and Behavioral Research, New York, New York, United States

## Abstract

Older adults with cognitive impairment (OAwCI) may be challenged by increasing demands to engage with technology to accomplish daily activities. We investigated technology attitudes, proficiency, and usage across health, social, transportation, leisure, and domestic domains among OAwCI, including those with mild cognitive impairment (MCI), traumatic brain injury (TBI), and post-stroke cognitive impairment (PSCI). We examined if age, gender, health, cognition, and attitude towards technology (comfort, interest, efficacy) predicted technology proficiency and use in each domain and technology usage patterns. Data were gathered from 159 OAwCI, age range 60-93 years who participated in the Everyday Needs Assessment for Cognitive Tasks (ENACT) study. We found that OAwCI were frequent users of technology, were largely proficient in the use of mobile devices, and had generally positive attitudes toward technology. With respect to use of technology, the data indicated that the highest degree of engagement was with social technologies (e.g., telephone, email, text) and the least engagement was in the use of technologies in the transportation domain (e.g., using apps to purchase tickets or schedule rides). Data from the regression analyses showed that younger age, more comfort and interest towards technology predicted more technology proficiency. Perceptions of memory problems predicted more proficiency. These findings underscore that OAwCi are able to and are receptive towards using technology. The findings also provide valuable insights for tailoring interventions to meet the needs and preferences of OAwCI.

